# Type 2 diabetes increases and metformin reduces total, colorectal, liver and pancreatic cancer incidences in Taiwanese: a representative population prospective cohort study of 800,000 individuals

**DOI:** 10.1186/1471-2407-11-20

**Published:** 2011-01-18

**Authors:** Meei-Shyuan Lee, Chih-Cheng Hsu, Mark L Wahlqvist, Hsin-Ni Tsai, Yu-Hung Chang, Yi-Chen Huang

**Affiliations:** 1 School of Public Health, National Defense Medical Center, Taipei, ROC; 2 Institute of Population Health Sciences, National Health Research Institutes, Zhunan, Taiwan, ROC

## Abstract

**Background:**

Metformin protection against cancer risk in Orientals is uncertain. We examined the possible metformin effect on total, esophageal, gastric, colorectal (CRC), hepatocellular (HCC) and pancreatic cancers in a Taiwanese cohort.

**Methods:**

A representative sample of 800,000 was drawn from the Taiwanese National Health Insurance data of 2000. A cohort of 480,984 participants 20 years or older, diabetes-cancer-free on 1st January 2000 was formed and categorized as four groups by DM and metformin usage status. Eligible incident cancer events had to occur one year after the index date until the end of 2007. The Cox proportional-hazards model evaluated relative risk of cancer for treated DM patients with or without metformin. The covariates included age, gender, other oral anti-hyperglycemic medication, Charlson comorbidity index (CCI) score and metformin exposure dosage and duration.

**Results:**

With diabetes but no anti-hyperglycemic medication, cancer incidence density increased at least 2-fold for total, CRC and HCC. On metformin, total, CRC and HCC incidences decreased to near non-diabetic levels but to varying degrees depending on gender and cancer type (CRC in women, liver in men). Adjustment for other oral anti-hyperglycemic agents usage and CCI made the benefit of metformin more evident [hazard ratios (95% confidence intervals): total 0.12 (0.08-0.19), CRC 0.36 (0.13-0.98), liver 0.06 (0.02-0.16), pancreas 0.15 (0.03-0.79)]. There was a significant gender interaction with metformin in CRC which favored women. Metformin dosage for a significant decrease in cancer incidence was ≤500 mg/day.

**Conclusions:**

Metformin can reduce the incidences of several gastroenterological cancers in treated diabetes.

## Background

Diabetes (DM) is a significant risk factor for total cancer incidence and mortality [1-3] and for some site-specific cancers, notably breast and colorectal (CRC) [4,5], and probably endometrial, prostate, and pancreas [6-8]. These cancers have possible underlying mechanisms to do with energy throughput and balance, hyperinsulinemia or insulin resistance, or other hormone sensitivity [9]. Such findings may be relevant even before DM is clinically manifest or biochemical criteria are met, which is to say with pre-DM or the metabolic syndrome.

Diabetes treatment might affect cancer incidence and mortality [8-13]. Since the 1960s, metformin (one of the biguanides) has become the first line anti-hyperglycemic agent in type 2 diabetes (T2DM) treatment worldwide [14]. It has been shown to be potentially cancer protective in a pilot study on incidence in Scotland [15], in a later cohort study [16] and, indirectly, in an administrative population-based study on mortality in Saskatchewan, Canada [17].

For site-specific cancers, the cohort by Libby et al [16] also provides evidence that CRC may be prevented by metformin; a retrospective cohort study of UK databases showed this for colorectal and pancreatic cancers [18]; and hospital-based case-control studies suggest this is so for hepatocellular in Italy [19] and pancreatic cancers in the US [7,8].

No information is available about these possibilities with regard to non-Caucasian ethnicity, especially where both T2DM prevalence is increasing and cancer patterns changing towards those in advanced economies, as in the Asia Pacific region [20-22]. Yet, there is little known about site-specific cancer in relation to metformin usage pattern (i.e., duration and dosage). Because the gastroenterological cancer cluster is the major cause of cancer in Taiwan [23], we used representative National Health Insurance (NHI) datasets for Taiwan to form a cohort to assess total and gastroenterological (esophagus, stomach, CRC, liver and pancreas) cancers incidences in relation to metformin usage among T2DM.

## Methods

### Data sources

Taiwan launched a single-payer NHI Program in 1995. As of 2007, 98.4% of Taiwan's 22.96 million population was enrolled in this program. The NHI Research Database (NHIRD) derived from this system, subject to a double scrambling protocol is provided for research purposes. Based on the registration files and original claims data for reimbursement in the NHIRD, specific data subsets are constructed [24].

### Cohort formation

The Longitudinal Health Insurance Database 2000 (LHID2000) contains all the original claims data of 800,000 beneficiaries, randomly sampled from the year 2000 Registry for Beneficiaries of the NHIRD [24]. There are no significant differences in the gender or age distributions or in the average insured payroll-related amounts between the patients in the LHID2000 and the original NHIRD. We have studied the LHID2000 with antecedent data from 1st January 1996, which allowed us to exclude people with prevalent DM during the period to 31st December 1999. To obtain incident T2DM subjects, we formed a cohort of 480,984 participants who were 20 years or older and were DM and cancer free on 1st January 2000 and non-use of DM medication from 1996-1999. The research was approved by the Institutional Review Board of the NHRI.

### Measurements

#### *T2DM ascertainment, grouping and index date*

T2DM patients were defined as those who had at least two DM records (A181, pre-ICD before 2000, or ICD-250) within one year during 2000-2007, followed up for at least six months, and had no insulin at any time except for brief usage during a hospital admission.

We categorized all cohort subjects into four groups. The first was the T2DM patients who used metformin (n = 12,005) during 2000-2007 with at least two prescriptions. The second was the T2DM patients who did not use metformin (n = 4,597) but had oral DM medications except metformin, defined in the same way as metformin users. The third was the T2DM patients who did not use any oral anti-hyperglycemic medication (n = 8,643). The last was those subjects without any diagnosis of DM or use of DM medications during 2000-2007 (n = 417,844).

The index date for a randomly-selected non-DM, corresponded to that of a metformin user, with the same gender-and-age (born in the same year and month). Those selected were required to have been followed for at least 6 months and cancer-free during the first year after their index date in the same way as their metformin user counterpart. To summarize, the index date was the date of first prescription of metformin (metformin users), that for the first prescription of any other oral medications (non-metformin users), that of first diagnosis of DM (DM without medication) or the assigned date for the non-DM group. Due to different index date assignments of those DM patients who did not have medication, we limited the consideration of any effect of metformin use to those who had oral anti-hyperglycemic medications.

#### Metformin usage

Mean metformin dose was the cumulative dosage of all metformin prescriptions to the event or censored date divided by the cumulative duration from the index date to the last metformin prescription before event or censored date or the end of 2007. Mean metformin dosage was expressed in daily 500 mg units. We treated 'duration' as a time-dependent variable in modeling to avoid immortal time bias [25].

#### Cancer event ascertainment and cancer incidence density

To consider an exposure (metformin) and outcome (cancer) relationship, incident cancer (A08-A14/ICD140-208) cases were only valid if they occurred at least one year after the index date until 31st December 2007. We only considered the first cancer in the second year or beyond and when cancer diagnosis was recorded a second time within any year. To be cancer free, there was no record of cancer at anytime after the index date. Otherwise, cancer status was regarded as uncertain and the subject deleted from the study. Cancers studied were those at 5 specific gastrointestinal sites were esophagus (150/A090), gastric (151/A091), CRC (153-154/A093-A094), hepatic (155/A095) and pancreatic (157/A096).

Cancer incidence density (CID) was calculated as the number of incident cancer events divided by 10,000 person-years at risk followed (years after the index date until the first cancer diagnosis before the end of 2007 or, for non-cancer subjects, date of withdrawal from NHI or the end of 2007). For comparison, we used the age-gender-adjusted incidence rates calculated from the 2007 National Cancer Registry in Taiwan.

#### Covariates

Comorbidity was measured by the Charlson comorbidity index (CCI) [26] using the diagnoses recorded in the NHI before the index date. This did not include the diagnosis of DM because subjects were DM-free before the index date. Use of any other oral anti-hyperglycemic agents (sulfonylureas, meglitinides, glitazones, and an alpha-glucosidase inhibitor) was recorded as 'yes' or 'no'. Other covariates included age (20-49, 50-59, 60-69, ≥70 yrs), gender and geographical region.

### Statistical analysis

The differences among four groups were evaluated by ANOVA (CCI score) and by chi-square (demographics). For the comparison of metformin users with those who had oral anti-hyperglycemic medications except metformin for total and 5 site-specific cancers, the hazard ratios (HRs) for cancer incidence were estimated by Cox proportional-hazards models. In these models, the time variable was the interval between the index date and the date of cancer ascertainment, or date of withdrawal from NHI, or December 31, 2007. The potential covariates included age, gender, other oral anti-hyperglycemic medication usage, CCI score and dose and duration of metformin exposure. Furthermore, gender-specific models were also evaluated for total and site-specific cancers. The proportional hazards assumption was evaluated by comparing estimated log-log survival curves for all covariates. The log-log survival plots, stratified by metformin usage status, of all models graphically showed two parallel lines, indicating no violation of the assumption. The statistics software SAS 9.1 was used for data management and modeling.

## Results

Since the non-DM subjects were sampled by matching to DM patients with metformin, the two groups have similar age, gender and geographical distribution. The majority were over 50 years old and males. More than 40% of the subjects lived in northern Taiwan and less than 5% lived in eastern Taiwan and off-shore isles. More females were in the DM without medication group (51.4%) than the others (45.1%-46.0%). The CCI median for the non-DM group indicates that more than half of them have no comorbidity with a mean of 0.25, but, for the DM groups, half experience 4 comorbidities or more. The follow-up time for four groups ranged between 0.5-7.99 years. The diabetes without metformin group had the shortest mean follow-up time (3.52 years) among groups. Around 2% DM patients withdrew from NHI, while this was more than 4% for the non-DM. For those who were metformin users, 30% subjects used ≤500 mg/day, half used metformin for more than 2 years. (Table 1)

**Table 1:** Characteristics of study subjects by diabetes and metformin using status

Descriptor	**Non-DM**^**†**^ (n = 10491)	**DM without medication**^**‡**^ (n = 8130)	**DM without metformin**^**§**^ (n = 4327)	**DM with metformin**¶ (n = 11390)	P-value
Age, n (%)					<0.001
20-49 yr	2685 (25.6)	2230 (27.4)	1067 (24.7)	2977 (26.1)	
50-59 yr	3483 (33.2)	2202 (27.1)	1156 (26.7)	3638 (31.9)	
60-69 yr	2406 (22.9)	1639 (20.2)	940 (21.7)	2530 (22.2)	
≥70 yr	1917 (18.3)	2059 (25.3)	1164 (26.9)	2245 (19.7)	
					
Female gender, n (%)	4822 (46.0)	4177 (51.4)	1966 (45.4)	5134 (45.1)	<0.001
					
Geographical area, n (%)					<0.001
North	4938 (47.1)	4145 (51.0)	1731 (40.0)	5031 (44.2)	
Central	2472 (23.6)	1739 (21.4)	1052 (24.3)	2638 (23.2)	
South	2761 (26.3)	2032 (25.0)	1359 (31.4)	3285 (28.8)	
East	250 (2.38)	150 (1.85)	138 (3.19)	357 (3.13)	
Off-shore	70 (0.67)	64 (0.79)	47 (1.09)	79 (0.69)	
					
Charlson comorbidity index score					<0.001
Mean ± SD	0.25 ± 1.28	5.29 ± 3.68	4.79 ± 3.77	4.38 ± 3.54	
Median ± IQR	0.00 ± 0.00	5.00 ± 6.00	4.00 ± 5.00	4.00 ± 6.00	
Min-Max	0-15	0-17	0-21	0-20	
					
Follow-up time (yr)					
Mean ± SD	3.94 ± 2.09	3.94 ± 2.14	3.52 ± 2.10	3.90 ± 2.08	
Median ± IQR	3.80 ± 3.49	3.80 ± 3.65	3.28 ± 3.47	3.76 ± 3.44	
Min-Max	0.50-7.99	0.50-7.99	0.50-7.99	0.50-7.99	
					
Withdrawal from NHI, n (%)	451 (4.30)	157 (1.93)	88 (2.03)	240 (2.11)	
					
Mean daily metformin dose, n (%)					
≤500 mg				3460 (30.4)	
500-1000 mg				4012 (35.2)	
≥1000 mg				3918 (34.4)	
					
Metformin use duration (day)					
Mean ± SD				931 ± 765	
Median ± IQR				755 ± 1200	
Min-Max				1-2911	

† Insured who had no diabetes (DM) and had not used diabetes medication during 1996-2007.

‡ Newly diagnosed DM patients who never used any DM medication during 2000-2007.

§ Newly diagnosed DM patients who never used insulin or metformin during 2000-2007.

¶ Newly diagnosed metformin-treated patients who never used insulin during 2000-2007.

Total and site-specific CIDs per 10000 person-years and 95% confidence intervals (CIs), as well as age-gender-standardized Taiwanese population cancer incidences, are listed in Table 2. Except for pancreatic cancer, both total and site-specific cancer standardized incidences are equivalent to the CIDs in our cohort. The total CID is 46.0 for non-DM subjects, 97.6 for DM patients without medication, 91.7 for DM patients without metformin and 44.8 for DM patients with metformin. Similar patterns were found regardless of gender.

**Table 2:** Cancer incidence density by diabetes and metformin using status

**Cancer type**^**†**^	**Taiwanese population Incidence**^**‡**^	Incidence density (95%CI) of study cohort (per 10,000 person-years)			
		
		Non DM (n = 10491)	DM without medication (n = 8130)	DM without metformin (n = 4327)	DM with metformin (n = 11390)
Total cancer	49.9	46.0 (39.5-52.6)^ab^	97.6 (86.9-108)^ac^	91.7 (76.6-107)^bd^	44.8 (38.6-51.0)^cd^
Male	59.3	50.3 (41.0-59.6)^ab^	122 (104-139)^ac^	107 (84.9-130)^bd^	47.7 (39.0-56.4)^cd^
Female	41.2	41.0 (31.9-50.1)^ab^	75.7 (62.6-88.8)^ac^	74.0 (54.1-93.8)^bd^	41.2 (32.4-50.1)^cd^
					
Esophagus	1.14	2.94 (1.28-4.60)	5.14 (2.62-7.66)	4.03 (0.81-7.26)	4.78 (2.74-6.83)
Male	2.09	3.63 (1.11-6.15)	7.45 (3.05-11.9)	5.09 (0.10-10.1)	5.83 (2.78-8.89)
Female	0.20	2.13 (0.04-4.21)	3.05 (0.38-5.73)	2.84 (-1.10-6.78)	3.52 (0.91-6.13)
					
Stomach	2.64	3.91 (1.99-5.83)	5.14 (2.62-7.66)	6.71 (2.55-10.9)	5.46 (3.28-7.65)
Male	3.49	3.63 (1.12-6.15)	6.78 (2.58-11.0)	8.90 (2.31-15.5)	4.58 (1.88-7.29)
female	1.86	4.23 (1.30-7.17)	3.67 (0.73-6.60)	4.26 (-0.56-9.09)	6.53 (2.98-10.1)
					
Colorectal	7.31	7.07 (4.50-9.64)^ab^	19.8 (14.9-24.8)^ac^	17.4 (10.7-24.1)^bd^	6.83 (4.39-9.27)^cd^
Male	8.63	6.78 (3.35-10.2)^a^	23.6 (15.8-31.4)^ab^	12.7 (4.83-20.6)	9.57 (5.66-13.5)^b^
female	6.06	7.40 (3.53-11.3)^a^	16.4 (10.2-22.6)^b^	22.6 (11.6-33.7)^ac^	3.52 (0.91-6.12)^bc^
					
Liver	6.55	5.88 (3.53-8.23)^ab^	24.3 (18.8-29.7)^ac^	18.7 (11.8-25.7)^b^	10.2 (7.24-13.2)^c^
Male	9.25	8.64 (4.76-12.5)^ab^	32.3 (23.2-41.4)^ac^	29.1 (17.2-41.0)^bd^	12.5 (8.01-16.9)^cd^
Female	3.90	2.65 (0.33-4.98)^a^	17.0 (10.7-23.3)^a^	7.10 (0.88-13.3)	7.54 (3.72-11.3)
					
Pancreas	0.99	2.45 (0.93-3.96)	5.14 (2.62-7.66)	4.70 (1.22-8.18)	4.78 (2.74-6.83)
Male	1.17	2.72 (0.54-4.90)	5.42 (1.67-9.18)	5.09 (0.10-10.1)	5.41 (2.47-8.36)
female	0.81	2.13 (0.04-4.21)	4.88 (1.50-8.27)	4.26 (-0.56-9.09)	4.02 (1.24-6.81)

† ICD-9-CM also incorporates the A-code for cancer which was used in Taiwan in ambulatory care settings before the year 2000. Total cancer (140-208/A08-A14), esophagus cancer (150/A090), stomach cancer (151/A091), colorectal cancer (153-154/A093-A094), liver cancer (155/A095), pancreas cancer (157/A096).

‡ Age-gender-standardized cancer incidence per 10,000 persons (95% confidence intervals) in the Taiwanese Cancer Registry Annual Report for 2007.

a, b, c, d: Statistical significance was estimated by using the overlap rule for 95% CIs. Where there is the same superscript it means that there is significant difference between the two groups.

For site-specific cancers, only CRC and liver cancer reveal significant differences among groups, which seem related to gender. For women, non-metformin using DM patients have three times the CRC CID of people without DM (22.6 vs. 7.4). Yet, compared to non-metformin users, users have only one-sixth of their counterparts' incidence (3.52 vs. 22.6). For liver cancer, it is only the difference in men among groups which achieves significance. The incidence rate for males, who have DM and do not use metformin (29.1) is three times and twice that of men without DM (8.6) and that of men with DM who use metformin (12.5), respectively.

The metformin users were less likely to develop all-type cancers, CRC and liver cancer (model 1, HRs, 0.47, 0.38 and 0.53, *P *< 0.01) than non-metformin users (Table 3). When adjusted for age, gender, CCI score and time-dependent duration of metformin use (Model 2), great reductions in cancer risk for metformin users are seen for total cancer, esophageal, liver, and pancreatic cancer (HRs, 0.13, 0.44, 0.06, and 0.15, respectively) though esophageal and CRC are not significant. With additional adjustment for use of other anti-hyperglycemic medication, the HR of metformin users to non-metformin users did not change substantially (Model 3). However, for CRC, the risk when metformin is used is smaller and significant (HR, 0.36, *P *< 0.05). No significant gender and metformin use interaction was found for total cancer and site-specific cancers except CRC (data not shown).

**Table 3:** Hazard ratios in metformin-treated type 2 diabetes for cancer incidence

Cancer type	cancer cases/total	**ID**^**†**^	**Model 1**^**‡**^ HR (95%CI)	**Model 2**^**§**^ HR (95%CI)	**Model 3**^**¶**^ HR (95%CI)
Total cancer					
Comparator^††^	140/4327	91.7	ref.	ref.	ref.
Metformin users	199/11390	44.8	0.47 (0.38-0.59)***	0.50 (0.40-0.62)***	0.12 (0.08-0.19)***
Esophagus cancer					
Comparator^††^	6/4193	4.03	ref.	ref.	ref.
Metformin users	21/11212	4.78	1.15 (0.46-2.84)	1.27 (0.51-3.16)	0.44 (0.07-2.61)
Stomach cancer					
Comparator^††^	10/4197	6.71	ref.	ref.	ref.
Metformin users	24/11215	5.46	0.78 (0.37-1.63)	0.86 (0.41-1.81)	1.41 (0.42-4.73)
Colorectal cancer					
Comparator^††^	26/4213	17.4	ref.	ref.	ref.
Metformin users	30/11221	6.83	0.38 (0.23-0.65)***	0.42 (0.25-0.71)**	0.36 (0.13-0.98)*
Liver cancer					
Comparator^††^	28/4215	18.7	ref.	ref.	ref.
Metformin users	45/11236	10.2	0.53 (0.33-0.85)**	0.54 (0.34-0.87)*	0.06 (0.02-0.16)***
Pancreas cancer					
Comparator^††^	7/4194	4.70	ref.	ref.	ref.
Metformin users	21/11212	4.78	0.99 (0.42-2.33)	1.11 (0.47-2.62)	0.15 (0.03-0.79)*

†: Incidence density per 10,000 person-years.

‡: Unadjusted hazard ratio, HR, (95% confidence interval, 95% CI), by Cox regression.

§: Adjusted for age group, and gender.

¶: Adjusted for age group, gender, other oral anti-hyperglycemic medication, CCI score, and duration of metformin exposure which was treated as a time dependent variable

††: Diabetes patients who had no metformin, but might have used any other oral anti-hyperglycemic medication.

*P < .05, **P < .01, ***P < .001

**Figure 1:**
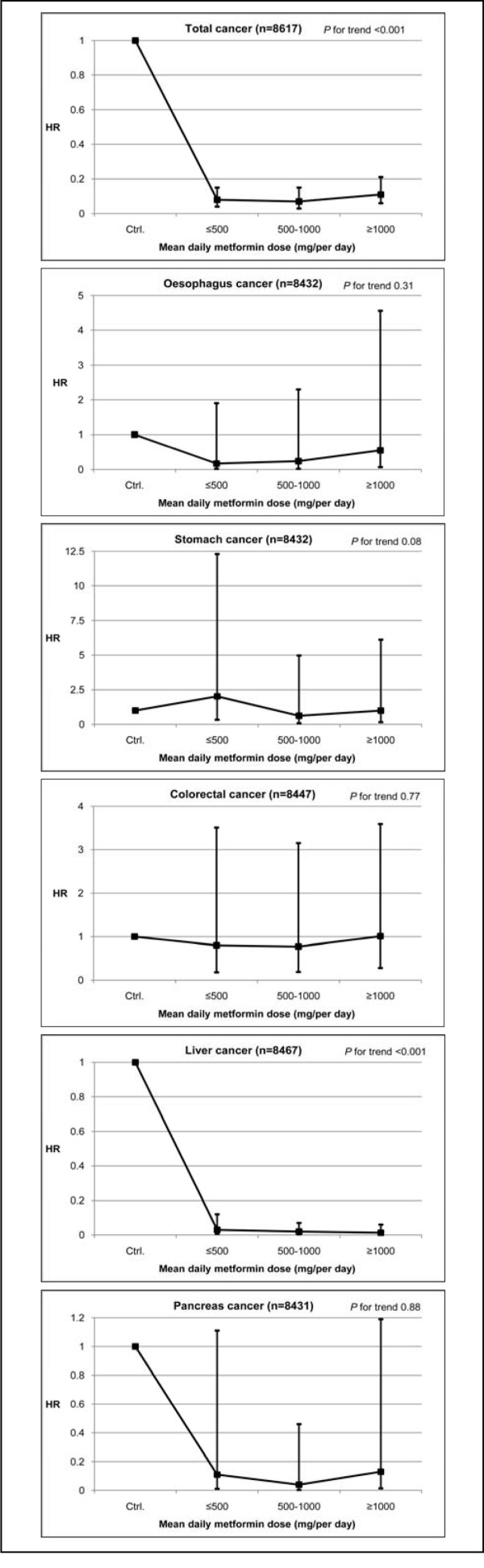
**Hazard ratios in metformin-treated type 2 diabetes for cancer incidence for men (n = 8,167)**. Adjusted for age group, gender, other oral anti-hyperglycemic medication, CCI score, and duration of metformin exposure (a time dependent variable). Reference: Diabetes patients use oral anti-hyperglycemic medication except metformin.

**Figure 2:**
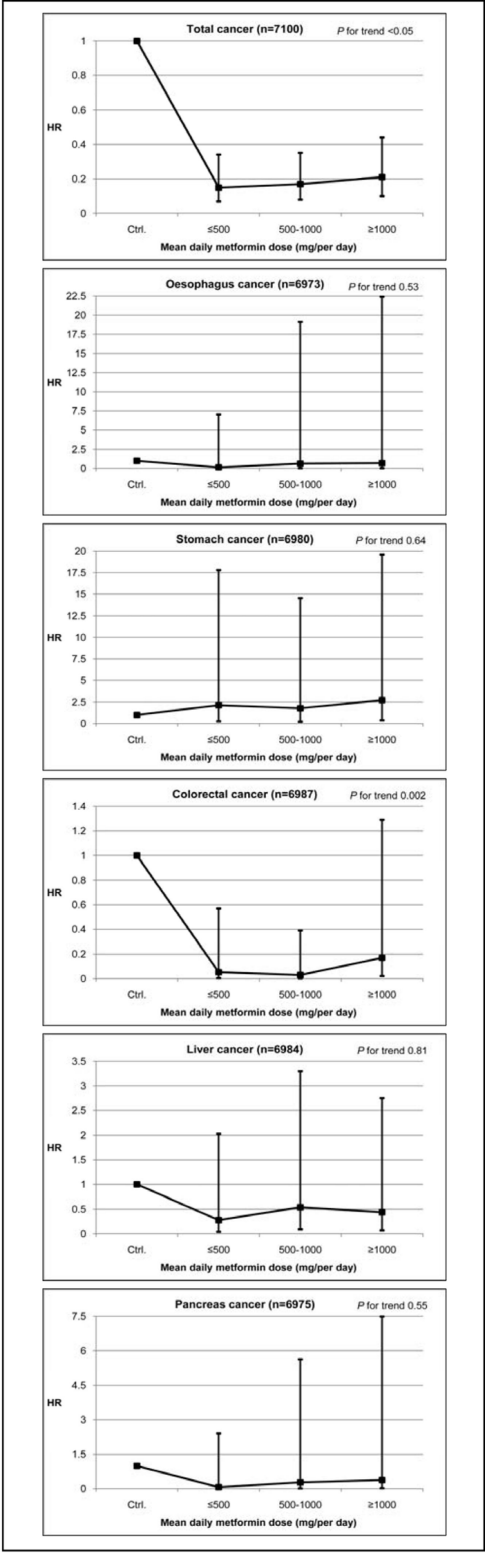
**Hazard ratios in metformin-treated type 2 diabetes for cancer incidence for women (n = 7,100)**. Adjusted for age group, gender, other oral anti-hyperglycemic medication, CCI score, and duration of metformin exposure (a time dependent variable). Reference: Diabetes patients use oral anti-hyperglycemic medication except metformin.

Metformin users were categorized into three groups by average daily doses compared to DM patients who use other oral medication by gender (Figures 1 and 2). Gender differences are apparent in CRC and liver cancer. In general, female metformin users are at a significantly lower risk of CRC for all three dosages compare to non-users (HRs, 0.05-0.17) while male users are not (HRs, 0.77-1.01). By contrast, there is an extremely low risk of liver cancer for male metformin users (HRs, 0.01-0.03), whereas there is a non-significant lower risk for females (HRs, 0.28-0.44) (data not shown). Cancer risk was significantly decreased with metformin, the dose can be as little as ≤500 mg per day, which may represent a threshold effect.

## Discussion

Among cancer free individuals, we found that total and all 5 site-specific gastroenterological cancer incidences increased with T2DM, although this was only significant for total, CRC and liver cancers. With metformin treatment, incident total cancer was reduced to near or below non-DM incidence. This was seen for both genders and at the lowest usual dose of metformin used in clinical practice (≤500 mg/day). This is unlikely to be due only to DM severity, because the dose-response is almost a plateau with higher doses, an indication of DM severity. In the case of CRC, the metformin-treated women have less cancer risk than the non-DM population. However, the interaction between gender and CRC suggests that some caution is required in the extrapolation of the findings for women to men. The same is not the case for gender and hepatocellular cancer (HCC), where, if women experience the higher incidence seen in men, they may benefit from metformin protection.

A shared pathogenesis for DM and certain cancers is possible, e.g., in beta oxidation of fatty acids or mitochondrial function. The prevention or management of the one might lead to corresponding outcomes for the other, as seems to be the case for metformin [8,12]. Metformin might protect against tumorigenesis through several mechanisms [8,10,11]. Metformin stimulates peripheral AMP-activated protein kinase (AMPK) with reduced hepatic gluconeogenesis, increased hepatic fatty acid oxidation and insulin sensitivity in muscle. In physiological circumstances, AMPK acts as an intracellular fuel sensor and is activated when the cellular AMP/ATP ratio increases. It is of interest to those cancers for which energy imbalance is a risk factor [22]. Dependent on cellular p53 and cyclin status, tumor cell cycle and growth is impaired by metformin. Metformin may counter age-related metabolic phenomena, such as sarcopenia, increased body fat and osteopenia, insulin sensitivity and adipokines. In our study, we have considered the role of age to any effect of metformin on the DM-cancer nexus; adjustment for age makes no discernable difference to the findings. Since the mechanism is likely to be through regulation of cellular energy metabolism, this has implications for measures designed to enhance energy expenditure by physical activity and avoid excess energy intake as well.

Severity and duration of DM is likely to be important for the effectiveness of metformin. In this administrative cohort, we do not have a direct measure of DM severity like glycated hemoglobin or of BMI. In Taiwan, the usual first line oral anti-hyperglycemic therapeutic agents for T2DM are sulfonylureas and biguanides (metformin). It can be inferred that, where DM is more difficult to control, the dose of one or both of these classes of oral anti-hyperglycemic agents will be increased. On the other hand, other anti-hyperglycemic agents may mask or mitigate against some of this metformin-dependent cancer protection. The study of Bowker et al. raises similar concerns about sulfonylureas [17] which may increase the risk of cancer in DM. We, therefore, considered dose and found it unlikely that increasing severity and its correction was the sole explanation for our findings. However, the finding that total and CRC CIDs may drop below that in the non-DM may still be attributed in part to the presence of unrecognized pre-DM. We also adjusted for other oral agent usage and found that this decreased the HR even further in the metformin users.

Since there are possible comorbidities which may modify the relation between DM, metformin and cancer, we further adjusted for CCI score in the analyses. The results were even more convincing after these adjustments and suggest that the comorbidities seen with DM may themselves be important in increasing cancer risk. The most likely candidate disease mechanism, common to most comorbidities, including obesity, would be inflammation which is one of the bases for cancer [21]. The CCI adjustment would have allowed for the more complete effect of metformin to be revealed.

A potential selection bias in this study comes from the prescription of metformin. On the one hand, those who were prescribed metformin might have been more likely to be obese, one of the well recognized cancer risks. So, the cancer protection effect of metformin illustrated in this study would be underestimated. On the other hand, those taking metformin might also have had their renal function better preserved (serum creatinine <1.5 mg/dL), which would have led to an underestimation of cancer risk incurred through severe kidney disease and an overestimation of metformin effect. Other DM treatment than oral agents could also affect risk of cancer in DM. In our study, the CIDs for total and site-specific cancers of those with DM but without medication were similar to those with DM but without metformin. We excluded patients who ever used insulin except briefly during a hospital admission. Sulfonylureas may increase risk of cancer [17], and we controlled for any other oral anti-hyperglycemic agents ever used before diagnosis of cancer.

Our findings are referable to the entire Taiwanese population and provided sufficient power to detect a metformin effect at several sites. The second Scottish study detected a favorable reduction in unadjusted CRC, breast and lung cancer incidences, and a significant adjusted CRC incidence, with metformin [15,16] in a population at high risk. Likewise, the Taiwanese population has a relatively high incidence of HCC and we report the first evidence to our knowledge that metformin use is linked to its reduction in the DM population. In the case of CRC, our report supports that of Libby et al for metformin in its prevention in Taiwanese women. Taiwan is experiencing increasing incidences of breast cancer and CRC [27] towards rates seen in European populations which may extend its relevance beyond Orientals.

We found a significant lower relative risk for pancreatic cancer in metformin users as have previous observations [6,7]. However, the CID in diabetes was not significantly different to that of non-DM. Li's study [7] which demonstrated potential pancreatic cancer protection by metformin, was hospital-based and case-control in the US. Ours was a representative population-base cohort of DM-cancer-free subjects at baseline. Our study resolves some of the outstanding issues in the US study in regard to generalizability and the possible confounding effects of other DM pharmacotherapy, other diseases and their treatment. However, ours is principally an oriental and Li's a mainly white population so that cross-ethnic robustness remains to be elucidated.

Metformin might operate to prevent or check tumorigenesis through a basic underlying mechanism and disorder, with generalizability. It may help sort out the pathogenetic, preventive and management differences for site-specific cancers. With incidence, the endpoint of our study, when a subject develops a cancer, this can be considered in relation to the oral anti-hyperglycemic agent used to that point and need not be confused by changes in medication subsequent to the diagnosis.

There are gender differences in the development of body fat distribution with relatively earlier and greater abdominal fatness (and fatty liver) in men [28] and in the protective role of estrogen against insulin resistance [29-33]. We found gender differences in the effect of metformin against CRC and HCC. While the prevalence of HCC and CRC is greater in men, women lose some protection against CRC after the menopause [34,35]. Where there has been a decline in CRC incidence in Canada, it has been mainly in women [36]. In Taiwan, hepatitis B and C with aflatoxin exposure is a more likely cause of cirrhosis and HCC than alcohol excess, although alcohol may be a contributor to the gender difference [27] and abdominal obesity through non-alcoholic steatohepatitis with cirrhosis may play a role. Gender disparity in HCC may be due to differences in MyD88-dependent IL-6 production [37]. Hepatic estrogen receptor status may also affect the progression of HCC [38]. In our study for CRC, metformin users were older than the others (data not shown), a somewhat elderly estrogen-deficient group. Nevertheless, insulin resistance is related to estrogen deficiency and/or androgen excess as evidenced in the polycystic ovarian syndrome and this is responsive to metformin [39,40]. Therefore, metformin might act differentially and favorably on estrogen-deficient women. The opposite may be the case in men where there is a gender difference in susceptibility to HCC along with altered estrogen metabolism and insulin resistance in cirrhosis [41].

The present study has several limitations. Firstly, we have required that for an eligible event to be counted it had to be diagnosed one year after metformin commenced. Consequently, for DM subjects and their non-exposure counterparts in the last year (2007), there would have been no chance to count their cancer outcome. Secondly, obesity is a risk factor for DM and cancer and should be considered as a confounder. However, this administrative dataset does not have systematic clinical measurements available for BMI or obesity. Body weight could differ in patients treated with different anti-DM drugs. This could lead to an underestimation of the true effect of metformin (the use of sulphonyloureas and gliazones is associated with weight gain and metformin with weight loss). While BMI data are not available, we can presume that the adverse outcomes of BMI outside acceptable limits will be manifest to some extent, in comorbidities. These we have taken into account in the CCI adjustment. Thirdly, for some cancers studied, the events were few and did not allow us to judge whether metformin is protective or not. Fourthly, we do not have direct linkage of the NHI database to death certificates. If a subject leaves the cohort, that death or migration is the likely reason. There were no differences in the disappearance rates among the three DM groups which might have led to erroneous interpretations of our data. In addition, there are caveats about populations to which extrapolations from our findings may be made. The population studied is dominantly Orientals. The problem of DM and its sequelae is nevertheless similar to that in Europeans [15-18]. At least for North East Asia, where living and eating patterns are similar, our large population findings should be relevant.

Metformin may reduce cancer risk and randomized-trials should be conducted. Our study provides evidence to encourage evaluation of the risk of cancer in DM patients. This is because the cohort evidence of DM antedating major cancers like those of breast and CRC, along with experimental, points to cause and effect. Moreover, our study shows that low dose metformin can prevent certain common cancers (CRC and HCC) and probably certain less common cancers (pancreas) for Taiwanese. Metformin has few adverse side effects; including that of lactic acidosis [42]. The gastrointestinal side effects are manageable if starting dose is low. Caution is required about effects on vitamin B-12 and homocysteine status and more work is required in large population studies to evaluate this [43].

## Conclusions

Low dose metformin is protective against total cancer and several cancers, including CRC in women, HCC in men and pancreatic for treated DM patients. This is evident in a population with an increasing prevalence of DM and at increasing risk of cancer profiles characteristic of economic development. Cancer prevention may be enhanced by avoiding DM or by its control with metformin. Since metformin would seem to act through cellular energy regulation, diet and exercise operate to reduce cancer risk in a similar way, although gender differences indicate that ancillary mechanisms and preventive and management approaches may be possible.

## Competing interests

The authors declare that they have no competing interests.

## Authors' contributions

MSL, CCH, MLW and YHC participated in developing the conceptual framework, in analysis, and interpretation, drafting, and approval of the final version of the report. HNT and YCH performed data analysis and approved the final version of the report.

## Pre-publication history

The pre-publication history for this paper can be accessed here:

http://www.biomedcentral.com/1471-2407/11/20/prepub
